# Exploration of the protective mechanisms of Icariin against cisplatin-induced renal cell damage in canines

**DOI:** 10.3389/fvets.2024.1331409

**Published:** 2024-02-21

**Authors:** Jiayi Liu, Liuwei Xie, He Zhai, Dongwei Wang, Xiao Li, Yao Wang, Mingqiang Song, Chao Xu

**Affiliations:** ^1^College of Police Dog Technology of Criminal Investigation Police University of China, Shenyang, China; ^2^Institute of Special Wild Economic Animals and Plants, Chinese Academy of Agricultural Sciences, Changchun, China

**Keywords:** Icariin, cisplatin, MDCK cells, apoptosis of cells, protection effect

## Abstract

This study delves into the protective mechanisms of Icariin (ICA) against cisplatin-induced damage in Madin-Darby canine kidney (MDCK) cells. Comprising two distinct phases, the investigation initially employed a single-factor randomized design to ascertain the minimal cisplatin concentration eliciting MDCK cell damage, spanning concentrations from 0 to 16 mmol/L. Concurrently, various concentrations of ICA (ranging from 5 to 50 mmol/L) were combined with 1 mmol/L cisplatin to determine the most efficacious treatment concentration. Subsequent investigations utilized four treatment groups: control, 1 mmol/L cisplatin, 1 mmol/L cisplatin + 20 mmol/L ICA, and 1 mmol/L cisplatin + 25 mmol/L ICA, aimed at elucidating ICA's protective mechanisms. Findings from the initial phase underscored a significant reduction in MDCK cell viability with 1 mmol/L cisplatin in comparison to the control (*P* < 0.01). Notably, the inclusion of 20 and 25 mmol/L ICA substantively ameliorated MDCK cell viability under 1 mmol/L cisplatin (*P* < 0.01). Moreover, cisplatin administration induced an elevation in inflammatory factors, malondialdehyde (MDA), reactive oxygen species (ROS), and Bax protein levels, while concurrently suppressing superoxide dismutase (SOD), catalase (CAT), and Bcl-2 expression (*P* < 0.01). Conversely, supplementation of 20 and 25 mmol/L ICA demonstrated a marked increase in mitochondrial membrane potential and levels of SOD, CAT, and Bcl-2 (*P* < 0.01). These interventions effectively attenuated inflammatory responses and suppressed Bax protein expression (*P* < 0.05), consequently mitigating cisplatin-induced apoptosis in MDCK cells (*P* < 0.01). In summary, these findings elucidate the role of ICA in impeding apoptosis in cisplatin-induced MDCK cells by regulating inflammatory responses, oxidative stress, and autophagic protein expression.

## 1 Introduction

Cisplatin is a widely utilized chemotherapeutic agent for the treatment of diverse cancer types and sarcomas. Its primary mode of action involves the formation of cross-links between purine bases within DNA, thereby disrupting DNA repair mechanisms in malignant cells, ultimately resulting in DNA damage and enhanced cellular apoptosis (1). However, the chemotherapy involving cisplatin and its derivatives can result in potent side effects, particularly causing inflammation, oxidative stress, and fibrosis in renal tubular cells, ultimately leading to acute kidney injury and even renal failure (2–4). Therefore, seeking a natural medicinal component to mitigate cisplatin-induced renal cell damage is of utmost importance. Icariin (ICA), the primary bioactive compound derived from the medicinal herb Epimedium, has been extensively utilized in the therapeutic management of systemic disorders including lupus nephritis, inflammatory bowel disease, rheumatoid arthritis, and cancer (5, 6). Research by Xie et al. (7) has demonstrated that ICA exhibits a potential to mitigate mortality and acute kidney injury in mice with sepsis induced by cecal ligation and puncture. This effect is achieved through the inhibition of renal oxidative damage, inflammatory responses, cell apoptosis, as well as improvement in vascular permeability. Additionally, investigations have unveiled the anti-fibrotic properties of ICA which are attributed to its ability to suppress the activation of renal fibroblasts via inhibiting signaling pathways associated with inflammatory cytokines IL-1β/TGF-β (8). Moreover, high doses of ICA have shown significant improvement in fibrosis, mitochondrial dynamics, and mitochondrial function in a rat model of chronic renal failure (CRF) (9). However, there is limited literature on ICA's potential in alleviating renal cell damage induced by cisplatin chemotherapy. Therefore, this study utilizes cisplatin-induced damage in Madin–Darby canine kidney (MDCK) cells as a model to investigate how ICA may alleviate oxidative stress and enhance mitochondrial activity to mitigate cisplatin-induced cell damage. This exploration seeks to identify natural adjuncts to cisplatin treatment, offering new insights into the clinical reduction of cisplatin-induced renal toxicity.

## 2 Materials and methods

### 2.1 Chemicals and reagents

Madin–Darby canine kidney cells (MDCK, Procell CL-0154) were obtained from Wuhan Procell Life Science and Technology Co., Ltd. Icariin (purity ≥ 99%) was purchased from Chengdu Mansite Biotechnology Co., Ltd. (A0049). DMEM culture medium (LV-DMEM001) and fetal bovine serum (WS500T) were all procured from the Liver Biotechnology (Shenzhen) Co., Ltd. Cisplatin (479306–1G) was purchased from Sigma-Aldrich in the United States. The PI/Annexin V cell apoptosis kit (P-CA-201) was obtained from BD Biosciences in the United States. Malondialdehyde (MDA, ml095226), catalase (CAT, ml095270), and superoxide dismutase (SOD, ml103496) assay kits were purchased from Shanghai Enzyme-linked Biotechnology Co., Ltd. Tumor necrosis factor α (TNF-α, E-EL-H0109c), interleukin-6 (IL-6, E-EL-H6165), and interleukin-1β (IL-1β, E-EL-H0149c) were procured from Jiangsu Zhengneng Biological Technology Co., Ltd.

### 2.2 Experimental design

#### 2.2.1 Screening of cisplatin cytotoxicity and Icariin treatment concentration for MDCK cells

This experiment was divided into two parts. In the first part, a single-factor completely randomized design was used, with the addition of cisplatin concentration in MDCK cell culture medium as the single variable. Seven concentrations (0 mmol/L, 0.5 mmol/L, 1 mmol/L, 2 mmol/L, 4 mmol/L, 8 mmol/L, 16 mmol/L) were set, each with 6 replicates, to determine the half-maximal inhibitory concentration (IC50) of cisplatin on MDCK cell viability. This information was used to select the modeling concentration for this experiment. In the second part, a single-factor completely randomized design was again used, with Icariin concentration in MDCK cell culture medium as the single variable. Seven concentrations (0 mmol/L, 5 mmol/L, 10 mmol/L, 15 mmol/L, 20 mmol/L, 25 mmol/L, 50 mmol/L) were set, each with 6 replicates, to screen for the effective treatment concentration of Icariin on MDCK cells following cisplatin treatment.

#### 2.2.2 Mechanism of Icariin treatment for MDCK cells post cisplatin induction

This experiment utilized a single-factor completely randomized design. Four treatment groups were established: normal culture of MDCK cells (control group), culture medium containing 1 mmol/L cisplatin, and 0, 20, 25 mmol/L of Icariin. Each treatment group had 6 replicates, aimed at investigating the mechanism of Icariin treatment on MDCK cells following cisplatin induction.

### 2.3 Cell culture

MDCK cells were seeded in DMEM culture medium and placed in a 37°C incubator with 5% CO_2_ for 24 h. The medium was changed every 12 h. When the cell density reached ~90%, cells in the logarithmic growth phase were digested and counted using a counting plate. About 9 × 10^3^ cells were seeded into each well of a 96-well cell culture plate. After 24 h of incubation, when the cells adhered to the wells, they were co-cultured with cisplatin at concentrations of 0, 0.5, 1, 2, 4, 8, and 16 mmol/L for 12 h. This was done to determine the concentration of cisplatin that achieves a half-maximal inhibitory effect on MDCK cell viability. Using the half-maximal inhibitory concentration of cisplatin, the cell culture process was repeated to obtain MDCK cells with impaired viability. These cells were then subjected to medium changes containing ICA at concentrations of 5 mmol/L, 10 mmol/L, 15 mmol/L, 20 mmol/L, 25 mmol/L, and 50 mmol/L. The survival rates of MDCK cells in each group were calculated.

### 2.4 Measurements

#### 2.4.1 Cell viability assay

For cells in logarithmic growth phase treated with cisplatin or Icariin as described in section 2.2.1, cell suspensions were prepared and counted. The cell suspensions (100 μL per well) were seeded into a 96-well plate. After pre-incubation in a cell culture incubator (37°C, 5% CO_2_) for 24 h, 10 μL of CCK-8 reagent was added to each well. The plate was then returned to the incubator for an additional 2 h. The absorbance (OD value) at 450 nm wavelength was measured using a microplate reader. The experiment was repeated three times, and the average of the results was taken as the final experimental outcome.

#### 2.4.2 Hoechst 33258 staining

Cell suspensions from each treatment described in section 1.2.2 were fixed with acetic acid-ethanol. They were washed with 0.01 mol/L PBS for 5 min. Subsequently, Hoechst 33258 working solution was used for staining, and the cells were left at room temperature for 15 min. After staining, the cells were washed with 0.01 mol/L PBS three times, with each wash lasting 5 min. Finally, the cells were sealed with a mixture of glycerol and PBS at a ratio of 1:9 or a water-soluble mounting medium, and observed under a fluorescence microscope.

#### 2.4.3 Mitochondrial membrane potential detection

The upper culture medium was aspirated from each treatment described in section 1.2.2. Then, 1 mL of prepared JC-1 staining working solution was added to each well, mixed gently, and incubated in the dark at 37°C for 20 min. After incubation, the cells were washed twice with staining buffer, and then removed, sealed with an anti-fluorescence quencher, and observed and photographed under a fluorescence microscope.

#### 2.4.4 Flow cytometry analysis

Cells from each treatment group described in section 1.2.2 were collected using a scraper. Cells from each well were resuspended in 100 μL of binding buffer, and 5 μL of FITC-Annexin V and 5 μL of PI working solution were added to each group. The cells were then incubated in the dark for 15 min at room temperature. After incubation, the cells were washed with binding buffer, centrifuged to remove the supernatant, and resuspended in 400 μL of binding buffer for flow cytometry analysis.

#### 2.4.5 Western blot analysis

The cells from each treatment group described in section 1.2.2 were lysed in RIPA lysis buffer on ice. After lysis, the samples were centrifuged at 3000 rpm for 5 min. Appropriate protein was added to buffer and boiled, followed by gel electrophoresis, membrane transfer, and blocking with 5% skim milk powder at 4°C overnight. The membrane was then incubated overnight with antibodies against Keap1-Nrf2 (HY-151362), HO-1 (HY-P70276), BAX (HY-P0081), and BCL-2 (HY-P72101) from Med Chem Express LLC. The next day, secondary antibodies were added, incubated at room temperature for 1.5 h, followed by washing. After washing, secondary antibodies were added, incubated at room temperature for 1 h, and ECL exposure was performed.

### 2.5 Statistical analysis

The formula for calculating cell viability is as follows:


$$\begin{array}{l} \text{Cell Viability }=\;\left(\text{As }-\text{ Ab}\right)/\;\left(\text{Ac }-\text{ Ab}\right)\;\times \;100\text{\%} \end{array}$$

in which As is the absorbance value of the experimental well (containing cells, culture medium, and CCK-8 reagent); Ab is the absorbance value of the blank well (containing culture medium and CCK-8 reagent); Ac is the absorbance value of the control well (containing cells, culture medium, and CCK-8 reagent).

Data underwent basic statistical analysis using the MEANS module in SAS 9.4. In Experiment 1, the GLM module performed variance analysis for canine MDCK cell viability under varying cisplatin concentrations and damaged cell viability under different ICA concentrations. Duncan's multiple comparison test identified the minimum cisplatin concentration inducing cell damage and the optimal ICA treatment concentration. In Experiment 2, the GLM module analyzed physiological indicators of canine MDCK cells under diverse treatments. Duncan's multiple comparison test elucidated the mechanism of ICA treatment on cell damage. A significance level of *P* < 0.05 was considered, and GraphPad Prism 8.0 software generated statistical graphs.

## 3 Results analysis

### 3.1 Cisplatin-induced cell damage and selection of optimal Icariin treatment concentration

**Figure 1:**
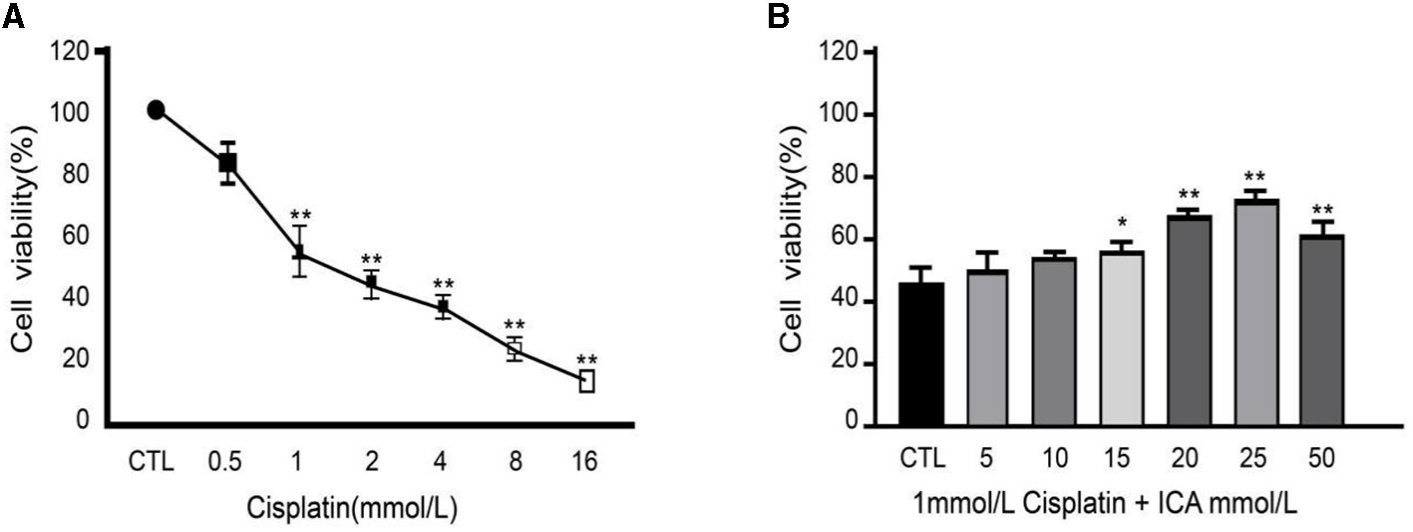
**(A, B)** Effect of Epimedium on therapeutic efficacy of cisplatin-induced damage to canine MDCK cells. ^*^Indicates a significant difference with *P* < 0.05; ^**^Indicates a highly significant difference with *P* < 0.01.

After 48 h of co-culture with culture medium containing 0, 0.5, 1, 2, 4, 8, or 16 mmol/L of cisplatin, it was observed that the viability of MDCK cells decreased significantly with the increase in cisplatin concentration (Figure 1A). Specifically, when the concentration of cisplatin in the culture medium reached 1 mmol/L, the viability of MDCK cells was significantly lower than that of the blank control group (*P* < 0.01), and cell viability dropped to 55%, approaching the half-inhibitory concentration. Based on these results, MDCK cells were co-cultured with culture medium containing 1 mmol/L cisplatin in addition to 0, 5, 10, 15, 20, 25, or 50 mmol/L of Icariin for 48 h. It was found that the viability of MDCK cells in the groups with 20, 25, and 50 mmol/L Icariin added to the culture medium was significantly higher than that in the control group and the groups with 5, 10, or 15 mmol/L of Icariin (*P* < 0.05) (Figure 1B). Among these concentrations, 20 and 25 mmol/L of Icariin showed the most significant treatment effect on canine MDCK cell viability. Therefore, in this study, 1 mmol/L of cisplatin concentration was selected as the toxic concentration for MDCK cells, and 20 and 25 mmol/L of Icariin were chosen as the treatment concentrations for MDCK cells after cisplatin exposure.

### 3.2 Effects of Icariin on the antioxidant and anti-inflammatory functions of MDCK cells induced by cisplatin

**Figure 2:**
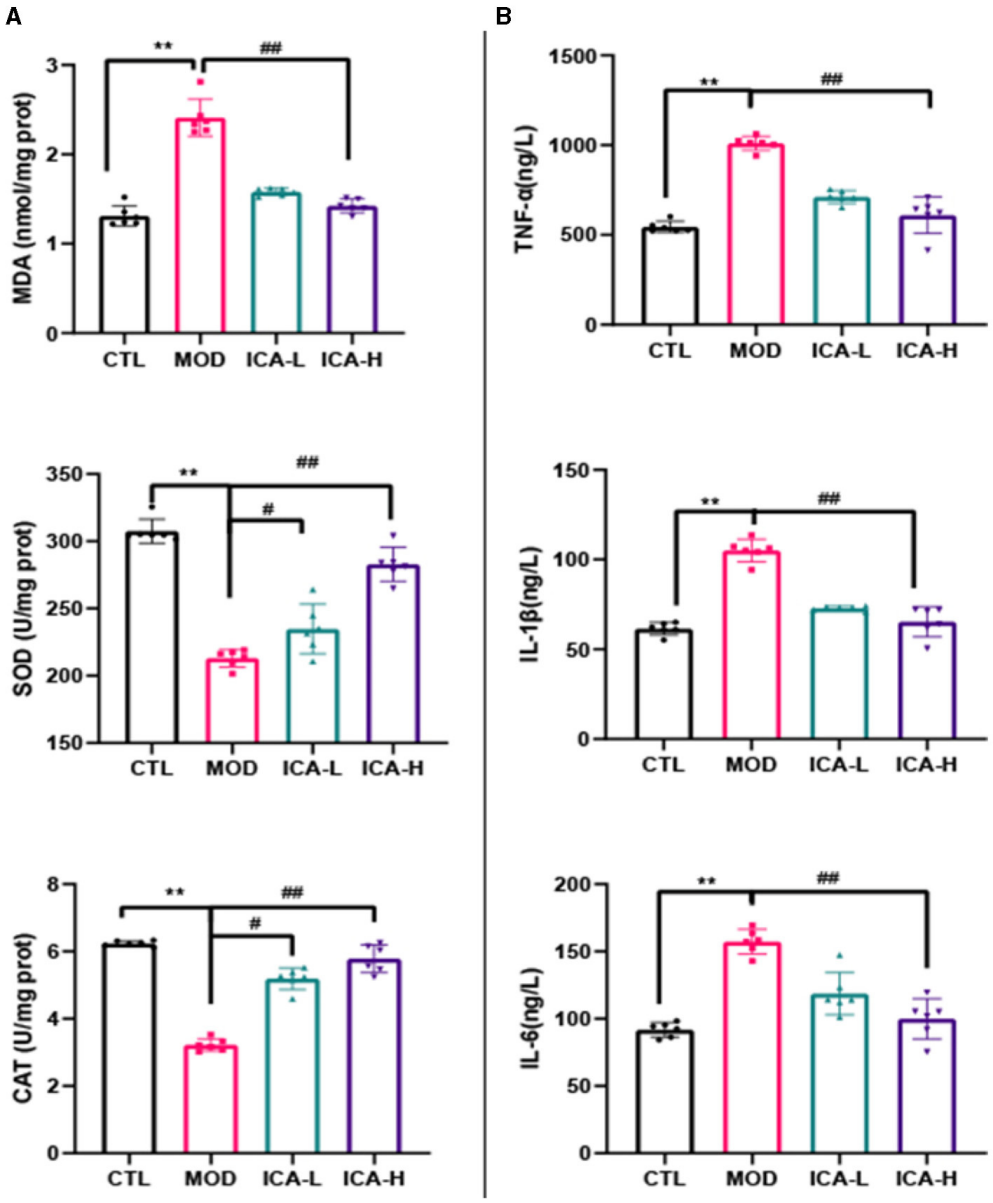
**(A, B)** Effect of Icariin on the antioxidant and anti-inflammatory functions of MDCK cells after cisplatin induction. ^*^, ^#^Both indicate significant differences; ^**^, ^*##*^Both indicate highly significant differences.

As shown in Figure 2A, the activity of SOD and CAT in canine MDCK cells treated with cisplatin (MOD group) was significantly lower than in the CTL control group (*P* < 0.01), while the MDA content was significantly higher than in the control group (*P* < 0.01). In comparison to the MOD group, the addition of 20 and 25 mmol/L Icariin to the culture medium significantly increased the activity of SOD and CAT in MDCK cells induced by cisplatin (*P* < 0.01) and significantly decreased the MDA content (*P* < 0.01). This indicates that Icariin effectively improves the antioxidant function of MDCK cells after cisplatin induction.

As shown in Figure 2B, the content of TNF-α, IL-1β, and IL-6 in canine MDCK cells treated with cisplatin in the culture medium (MOD group) was significantly higher than in the CTL control group (*P* < 0.01). However, in comparison to the MOD group, the addition of 20 and 25 mmol/L Icariin to the culture medium effectively inhibited the production of TNF-α, IL-1β, and IL-6 in MDCK cells induced by cisplatin (*P* < 0.01). This indicates that Icariin effectively enhances the anti-inflammatory capability of MDCK cells after cisplatin induction, and the effectiveness of its action is dependent on the dose of Icariin.

### 3.3 Effects of Icariin on the expression of autophagy proteins in MDCK cells induced by cisplatin

**Figure 3:**
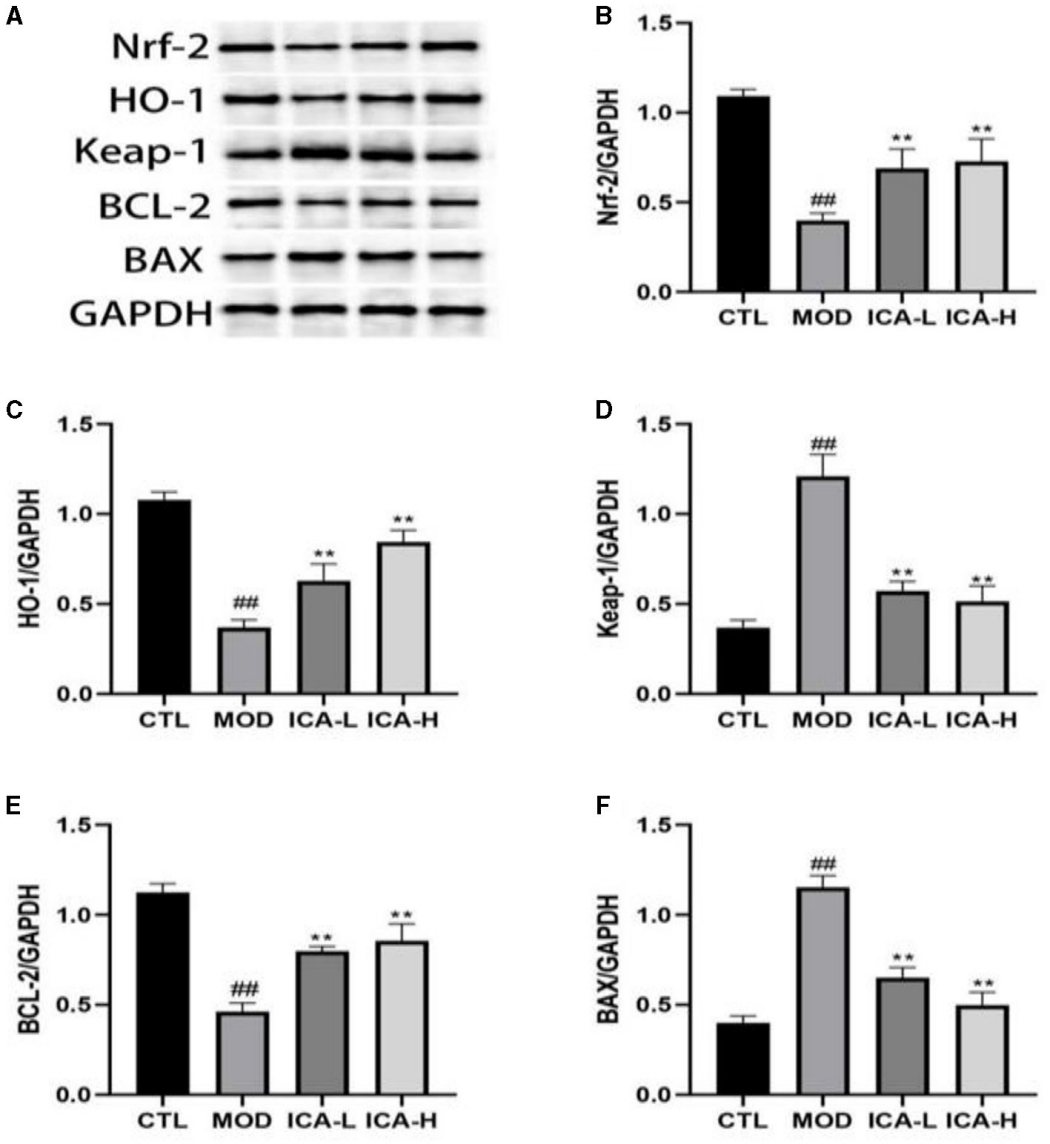
**(A–F)** Effect of Icariin on the expression of autophagy proteins in MDCK cells after cisplatin induction. ^*^, ^#^Both indicate significant differences; ^**^, ^*##*^Both indicate highly significant differences.

The protein immunoblot results (Figure 3) show that the expression of Keap-1 and Bax proteins in canine MDCK cells treated with cisplatin in the culture medium (MOD group) was higher than in the CTL control group (*P* < 0.01). Conversely, the expression of Nrf-2, HO-1, and Bcl-2 proteins showed the opposite trend (*P* < 0.01). In comparison to the MOD group, the addition of 20 and 25 mmol/L Icariin to the culture medium effectively inhibited the expression of Keap-1 and Bax proteins in MDCK cells induced by cisplatin (*P* < 0.01) and promoted the expression of Nrf-2, HO-1, and Bcl-2 proteins (*P* < 0.01). This indicates that ICA can significantly enhance the autophagy function of canine MDCK cells after cisplatin induction.

### 3.4 Effects of Icariin on mitochondrial membrane potential and nuclear apoptosis in MDCK cells induced by cisplatin

**Figure 4:**
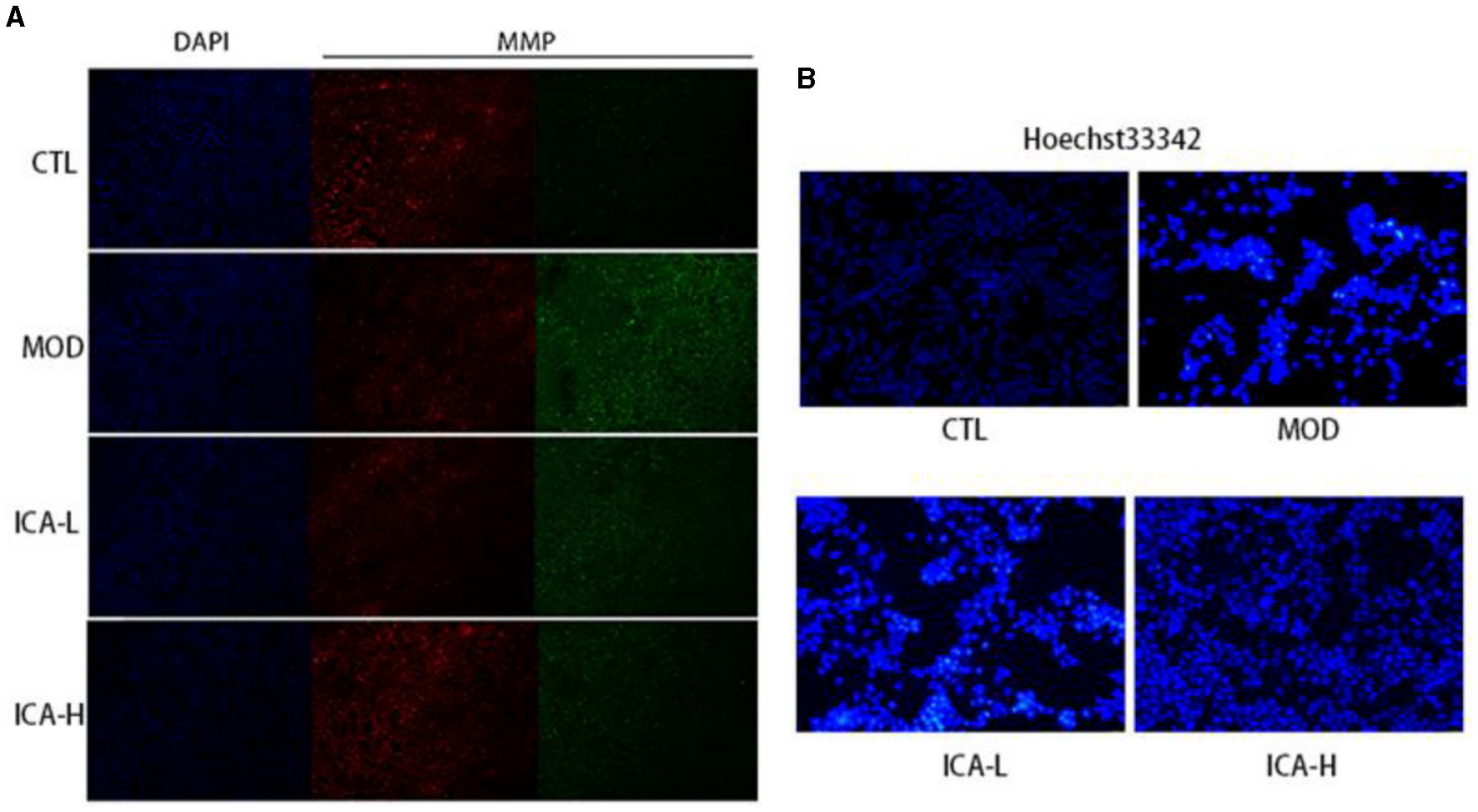
**(A, B)** Effect of Icariin on mitochondrial membrane potential and nuclear apoptosis in MDCK cells induced by cisplatin.

After JC-1 staining of MDCK cells, the ratio of red to green fluorescence can indicate changes in mitochondrial membrane potential. As shown in Figure 4A, compared to the CTL control group, the cells in the cisplatin (MOD group) displayed significantly increased green fluorescence and reduced red fluorescence, indicating that cisplatin cultivation reduced the mitochondrial membrane potential in MDCK cells. In comparison to the MOD group, the administration of 20 and 25 mmol/L Icariin reduced green fluorescence and significantly increased red fluorescence in MDCK cells. This suggests that Icariin improves the mitochondrial membrane potential in MDCK cells induced by cisplatin. Furthermore, compared to the CTL control group (Figure 4B), MDCK cells in the MOD group displayed enhanced nuclear staining, with brighter fluorescence and a round or shrunken, clustered appearance. In contrast, compared to the MOD group, the administration of 20 and 25 mmol/L Icariin reduced the number of bright spots in nuclear staining, and this effect appeared to be dose-dependent. This indicates that Icariin effectively inhibits apoptosis in MDCK cells induced by cisplatin.

### 3.5 Effects of Icariin on apoptosis in MDCK cells induced by cisplatin

**Figure 5:**
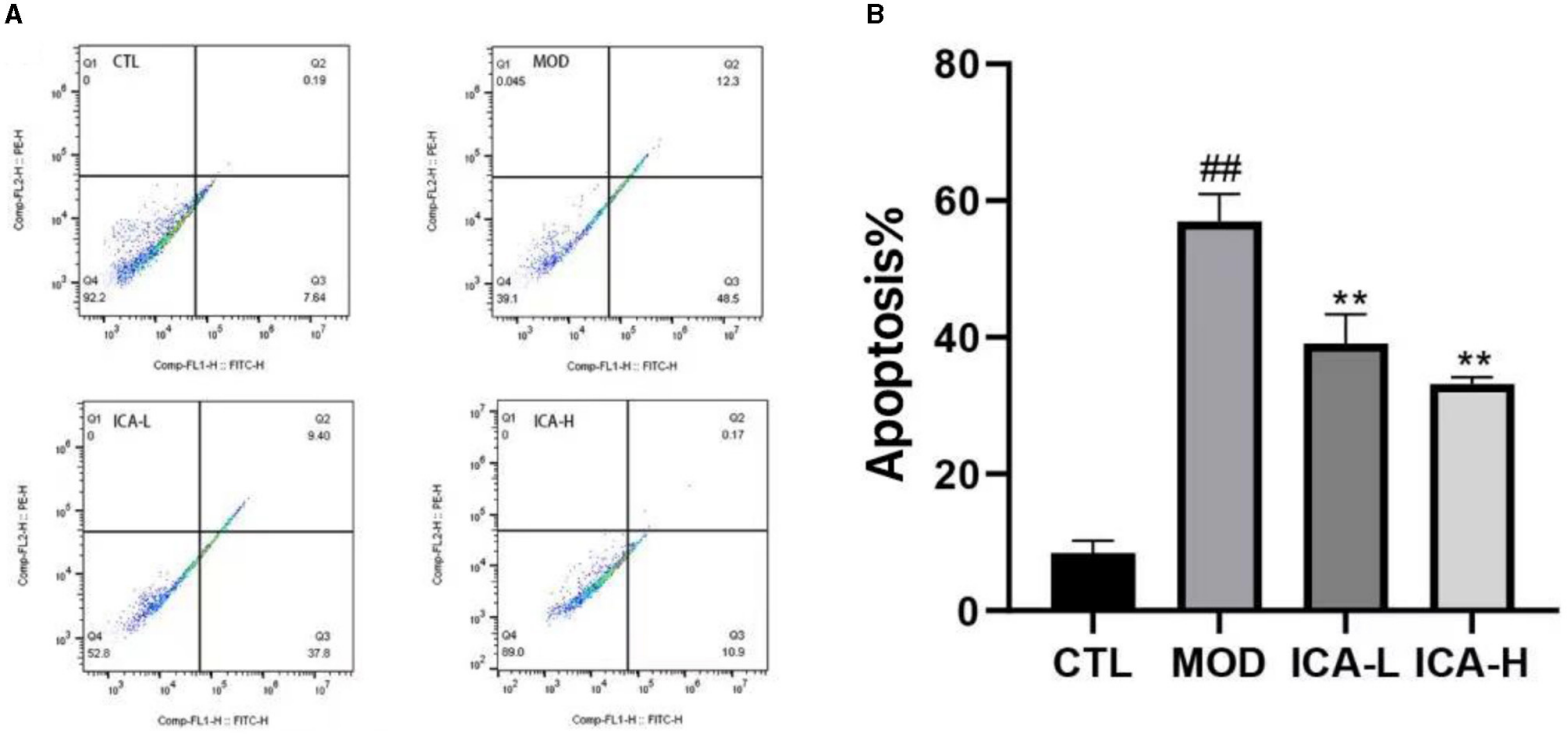
**(A, B)** Effect of Icariin on apoptosis of MDCK cells after cisplatin induction.

The results of apoptosis detected by flow cytometry in MDCK cells are shown in Figure 5. The apoptosis rate of MDCK cells in the cisplatin-added culture medium (MOD group) was significantly higher than in the CTL control group (*P* < 0.01). However, when compared to the MOD group, the addition of 20 and 25 mmol/L Icariin significantly increased the cell survival rate of MDCK cells induced by cisplatin (*P* < 0.01), and this treatment effect exhibited a dose-dependent relationship. This indicates that Icariin effectively enhances the viability of MDCK cells induced by cisplatin.

## 4 Discussion

The use of cisplatin in the treatment of various forms of cancer and sarcomas remains one of the most effective approaches, primarily by impeding DNA repair mechanisms in aberrant cells, thereby fundamentally inhibiting cell proliferation (1, 10). However, cisplatin chemotherapy elicits potent adverse effects on the organism, notably causing renal tubular cell inflammation, oxidative stress, and fibrosis, consequently leading to acute kidney injury and even renal failure (2–4). Therefore, the quest for a natural therapeutic component to counter cisplatin-induced renal cell damage is of paramount importance. Studies have demonstrated that ICA possesses the capacity to mitigate renal oxidative damage, inflammatory responses, and cell apoptosis and is widely employed in the treatment of systemic diseases such as lupus nephritis, inflammatory bowel disease, rheumatoid arthritis, and cancer (5, 7). This study utilized a cisplatin-induced canine MDCK cell damage model to explore the mechanisms of ICA in alleviating cellular damage. Furthermore, the current research focus is on enhancing the scope and intensity of cisplatin treatment through the combination of cisplatin with natural therapeutic components (11, 12). Therefore, the search for natural adjuvants to be used in conjunction with cisplatin to alleviate its chemotherapy-induced side effects in clinical practice offers a new perspective. In this study, canine MDCK cells were co-cultured with cisplatin at varying concentrations, revealing a sharp decline in cell viability as cisplatin concentration increased. When the cisplatin concentration in the cell culture medium reached 1 mmol/L, the apoptosis rate of MDCK cells reached 53.4%. This underscores the considerable cellular damage caused by cisplatin treatment. Subsequently, the co-culture of canine MDCK cells with different concentrations of ICA in combination with 1 mmol/L cisplatin demonstrated a significant improvement in cisplatin-induced cell damage, particularly at concentrations of 20 and 25 mmol/L. Hence, in this study, the selected 1 mmol/L cisplatin concentration served as the cytotoxic concentration for MDCK cells, while 20 and 25 mmol/L ICA were chosen as the therapeutic concentrations for damaged MDCK cells.

Cells, as autonomous entities, boast an elaborate antioxidative system (13). Notably, oxidative stress and intracellular inflammation constitute key factors contributing to apoptosis. In cases of cellular damage, oxidative metabolism indicators provide a lucid representation of cell vitality. Gu et al. (14) investigation established that acute kidney injury induced by cisplatin administration in murine models exhibits distinct pathological traits, notably featuring oxidative stress, renal tubular cell apoptosis, and inflammatory responses. This finding aligns with the research conducted by Li et al. (15), where cisplatin was shown to induce dysregulation in oxidative parameters, including MDA, CAT, and SOD, alongside the disturbance of inflammatory indicators such as TNF-α and IL-1β within murine renal cells. These perturbations expedite the process of cellular apoptosis, ultimately leading to the development of acute kidney injury in mice. Similar patterns were observed in this study, where cisplatin exhibited analogous effects. It reduced the activities of SOD and CAT in canine MDCK cells while elevating the levels of MDA, TNF-α, IL-1β, and IL-6. It is evident that cisplatin's mechanism for inducing cell apoptosis is consistent and universally applicable, with the alleviation of cisplatin chemotherapy side effects achievable through the control of cellular oxidative stress and inflammatory reactions. This inference has garnered recognition from numerous researchers. Studies have shown that antioxidants such as epigallocatechin gallate (16), hesperidin (17), and quercetin (2) can ameliorate cisplatin-induced oxidative stress and inflammation, thus mitigating cellular apoptosis during cisplatin chemotherapy. The subject of this study, Icariin (ICA), exhibits similar properties. The addition of 20 mmol/L−25 mmol/L ICA to cell culture media effectively enhances cellular anti-inflammatory and antioxidant capabilities, resulting in a more than 45% increase in cell survival rates. This suggests that ICA holds promise as an adjunct therapy with cisplatin, providing a pharmacological basis for alleviating cisplatin chemotherapy side effects in clinical practice.

Oxidative stress within cells primarily manifests in the mitochondrial energy metabolism process. When the intracellular antioxidant system fails to counteract the ROS generated by mitochondrial bio-oxidation, it leads to mitochondrial damage (18, 19). The resulting insufficient energy supply fails to sustain cellular metabolism, ultimately triggering cell apoptosis. In this study, it was found that cisplatin induces a reduction in mitochondrial membrane potential in canine MDCK cells, leading to mitochondrial damage, which may be one of the significant reasons for inducing cell apoptosis. Additionally, the content of intracellular antioxidants determines the extent of the oxidative stress response in cells. Oxidative regulatory proteins such as Nrf-2, Keap1, BCL-2, and BAX mutually promote and inhibit each other, collectively maintaining the normal operation of intracellular oxidative reactions (20, 21). Research by Wang et al. (22) demonstrated that Keap1, in its normal state, can bind to the antioxidant Nrf-2 and promote its degradation, while the overexpression of BAX can counteract the protective effect of BCL-2, pushing cells toward apoptosis. In this study, cisplatin treatment promoted the expression of Keap1 and BAX proteins in cells, leading to a decrease in the content of intracellular antioxidants Nrf-2 and BCL-2, thereby causing cellular oxidative stress. Icariin (ICA) effectively rebalances the expression of damaged cell antioxidant factors and mitochondrial membrane potential, thus offering a proactive approach to mitigate cisplatin-induced cellular apoptosis. This aligns with the findings of Shao et al. (23), where ICA activated the Nrf-2/HO-1 pathway, facilitating mitochondrial autophagy, alleviating oxidative-reductive imbalances and mitochondrial dysfunction, ultimately enhancing cell survival rates. Based on current research results, ICA appears to effectively ameliorate oxidative stress and cell apoptosis induced by cisplatin treatment in canine MDCK cells at the cellular level. However, validation experiments at the canine organism level require further investigation.

## 5 Conclusion

Our findings suggest that 20 and 25 mmol/L of ICA effectively alleviate cisplatin-induced damage in canine MDCK cells at the cellular level. This improvement is primarily attributed to the upregulation of intracellular antioxidative enzyme activity, downregulation of inflammatory responses, reduction in reactive oxygen species accumulation, inhibition of mitochondrial oxidative phosphorylation processes, and ultimately suppression of the cellular apoptosis pathway. These outcomes collectively mitigate cisplatin-induced damage on MDCK cells.

## Data availability statement

The original contributions presented in the study are included in the article/supplementary material, further inquiries can be directed to the corresponding author.

## Ethics statement

Ethical approval was not required for the studies on animals in accordance with the local legislation and institutional requirements because only commercially available established cell lines were used.

## Author contributions

JL: Conceptualization, Project administration, Writing—original draft. LX: Methodology, Writing—review & editing. HZ: Writing—original draft. DW: Methodology, Writing—original draft. XL: Methodology, Writing—original draft. YW: Project administration, Writing—original draft. MS: Formal analysis, Writing—review & editing. CX: Conceptualization, Writing— review & editing.
